# Time Course of the Effects of Buxin Yishen Decoction in Promoting Heart Function and Inhibiting the Progression of Renal Fibrosis in Myocardial Infarction Caused Type 2 Cardiorenal Syndrome Rats

**DOI:** 10.3389/fphar.2019.01267

**Published:** 2019-10-23

**Authors:** Qi Qiu, Jinglin Cao, Yong Wang, Yunnan Zhang, Yun Wei, Xiaoyan Hao, Yu Mu, Yang Lin

**Affiliations:** ^1^Department of Pharmacy, Beijing Anzhen Hospital, Capital Medical University, Beijing, China; ^2^Lifescience School, Beijing University of Chinese Medicine, Beijing, China; ^3^Department of Ultrasound, Beijing Anzhen Hospital, Capital Medical University, Beijing, China; ^4^Department of Echocardiography, Beijing Anzhen Hospital, Capital Medical University, Beijing, China

**Keywords:** Buxin Yinshen decoction, cardiorenal syndrome, renal fibrosis, heart function, connective tissue growth factor

## Abstract

This study aimed to investigate the therapeutic effect of traditional Chinese medicine-Buxin Yishen decoction (BXYS) on type 2 cardiorenal syndrome (CRS) caused by myocardial infarction and explore the possible mechanism BXYS works. A chronic heart failure (CHF) rat model induced by left anterior descending coronary artery ligation was used and five groups were created that included a sham group, a CHF model group, a fosinopril group, a BXYS (0.4 g/kg) group and a BXYS (0.8 g/kg) group. Heart function, renal hemodynamics, neuroendocrine factors, serum, and urine concentration of soluble form connective tissue growth factor (sCTGF), expression of CTGF mRNA, CTGF, α-smooth muscle actin (α-SMA), and low-density lipoprotein receptor-related protein (LRP) in renal tissues were evaluated after 28 days and 60 days of drug administration. Histological analysis of kidney tissues was also performed. *In vitro* experiments were designed to verify the results of *in vivo* experiments by detecting factors including CTGF, α-SMA, in NRK-52E cells. Rats with CHF showed obvious pathophysiological changes including: altered renal hemodynamic parameters; dysregulated heart function; changes to serum concentrations of angiotensin II (AngII), cyclic guanosine monophosphate (cGMP), serum creatinine (Scr), blood urea nitrogen (BUN), C-reactive protein (CRP), brain natriuretic peptide (BNP); high serum and urine sCTGF concentration; high CTGF mRNA, CTGF, α-SMA and LRP expression in renal tissues; increased extracellular matrix (ECM) deposition and fibrosis in renal tissues. Treatment of BXYS was correlated with a restoration of heart function and improvement of renal hemodynamics, lower serum and urine sCTGF, lower CTGF mRNA, CTGF, α-SMA and LRP expression in renal tissues and lower ECM deposition. In addition, *in vitro* experiments showed that treatment with BXYS reduced the α-SMA and LRP concentration in NRK-52E cells, which were similar *in vivo* experiments. In conclusion, the current study provided evidences that BXYS played a role in improving heart function and delaying the progress of renal fibrosis. Meanwhile, the CTGF-LRP pathway might be one of the therapeutic targets for myocardial infarction caused type 2 CRS which showed a positive change after BXYS treatment and is worthy of further exploration.

## Introduction

Cardiovascular disease (CVD)-induced heart failure is the leading cause of mortality worldwide (Roth et al., 2015). Among people with CVD-induced heart failure, about 40%–50% also have chronic renal dysfunction, a situation known as cardiorenal syndrome (CRS) (McAlister et al., 2004; Van Deursen et al., 2014). Of the five subtypes of CRS (Ronco et al., 2008), type 2 CRS is described as chronic cardiorenal syndrome, which refers to progressive, chronic kidney disease caused by chronic cardiac dysfunction. Current treatment options for type 2 CRS include diuretics, angiotensin converting enzyme inhibitors (ACEIs), angiotensin receptor blockers, β-blockers, vasodilators, inotropic drugs, erythropoiesis-stimulating agents, vasopressin receptor 2 antagonists, and dialysis (Naranjo et al., 2017). However, many of these drugs target heart failure (HF) and can worsen renal function. Some drugs are contraindicated due to decreased efficacy and increased adverse reactions in patients with renal dysfunction. Identifying novel mechanisms in the pathophysiology of type 2 CRS, as well as a therapeutic strategy, remain major challenges in clinical practice.

Notably, renal interstitial fibrosis caused by chronic heart failure (CHF) is an important pathological feature of type 2 CRS nephropathy (Jois and Mebazaa, 2012), which often develops into end-stage renal disease that results in uremia and requires kidney transplant. Therefore, early prevention and treatment of CHF-induced renal interstitial fibrosis, including measures to improve cardiac function, are of great importance for improving the prognosis of type 2 CRS. Connective tissue growth factor (CTGF) has been reported in both cardiac and renal fibrosis studies as a key player in the process of fibrosis (Gravning et al., 2012; Zambrano et al., 2014). CTGF expression is high in tissues stimulated by angiotensin II (AngII), endothelin-1 (ET-1), reactive oxygen species (ROS), mechanical tension, hypoxia and other factors (Matsui and Sadoshima, 2004). Activated CTGF can promote the synthesis of extracellular matrix (ECM), including type I and III collagen and fibronectin (FN). It also contributes to cell chemotaxis, proliferation, regulation of apoptosis, angiogenesis, and renal fibrosis (Matsui and Sadoshima, 2004). Current studies have shown that low density lipoprotein receptor-related protein (LRP) is widely expressed as a receptor for CTGF in mesangial cells, renal tubular epithelial cells and renal tubulointerstitial cells (Mason, 2013). The combination of CTGF and LRP may trigger a series of signal transduction events including tyrosine residue phosphorylation in the C-terminus of the LRP protein, activation of intracellular signaling molecules, and transcription of target genes. These signal transduction events may induce phenotypic transformation of renal cells causing glomerulosclerosis and kidney interstitial fibrosis (Mason, 2013).

Previous studies have reported that Yixin Jiedu (YXJD) formula could significantly improve cardiac function of left anterior descending coronary artery (LAD)-ligated rats and delay the process of cardiac fibrosis simultaneously (Li et al., 2012a; Li et al., 2012b). Buxin Yishen decoction (BXYS) is a Chinese clinical formula, which is based on YXJD and added *Poria* [*Poria cocos* (Schw.) Wolf] in it to enhance its effect on renal protection. BXYS consists of six herbal medicines, *Astragali Radix* [*Astragalus membranaceus* (Fisch.) Bunge var. *mongholicus* (Bunge) Hsiao, 37.5%], *Aconiti Lateralis Radix Praeparata* (*Aconitum carmichaeli* Debx., 11.25%), *Salviae Miltiorrhizae Radix Et Rhizoma* (*Salvia miltiorrhiza Bunge*, 18.75%), *Lonicerae Japonicae Flos* (*Lonicera japonica* Thunb., 12.5%), *Glycyrrhizae Radix Et Rhizoma* (*Glycyrrhiza uralensis* Fisch., 7.5%), *Poria* [*Poria cocos* (Schw.) Wolf, 12.5%]. However, its prevention and treatment of CRS renal interstitial fibrosis needs further study.

In the present study, a rat model of CHF, induced by LAD ligation was used to observe renal hemodynamic parameters and the process of renal tissue fibrosis dynamically. The expression of CTGF was analyzed to explore its role in CRS. The effect of BXYS on the expression of renal related molecular biological indicators, such as CTGF, and the process of renal fibrosis was explored. The results of *in vivo* experiments were verified by *in vitro* experiments.

## Materials and Methods

### *In Vivo* Experiments

#### Animal Care and Use

One hundred thirty male Sprague Dawley (SD) rats in specific pathogen-free grade weighing of 220 ± 10 g were purchased from Beijing Vital River Laboratory Animal Technology Co., Ltd. (license No. SCXK2016-0002). Rats were housed in temperature (23°C ± 2°C) and humidity (40% ± 5%) controlled rooms with a 12/12 light/dark cycle.

#### BXYS Decoction Preparation

Herbs consisted in BXYS were purchased from Beijing Tongrentang Pharmacy Co., Ltd. (Beijing, China) and prepared as described previously (Wang et al., 2012). In the process, dextrin was used as excipent at the ratio of 1:1 to mix with herbs extracts. And then BXYS was analyzed by LC-HRMS using ThermoFisher Scientific (TF, Dreieich, Germany) Dionex UltiMate 3000 (**Supplemental Figure 1**). Samples we used were identified (Voucher numbers: HQ-2016-007; JYH-2016-009; FZ-2016-011; GC-2016-012; DS-2016-013; FL-2016-014). BXYS decoction was prepared in two concentrations at 0.4 g/kg (equivalent to 1× human recommended dose, about 3.87 g/60 kg) and 0.8 g/kg (equivalent to 2× human recommended dose, about 7.74 g/60 kg).

#### Groups and Surgical Protocol

After 1 week’s adaptive feeding, 130 rats were randomly divided into sham operation group (30 cases) and model group (100 cases) according to body weight. The rats in model group underwent surgery of LAD ligation as described in previous study (Li et al., 2014). After surgery, as soon as the restoration of spontaneous respiration, rats were extubated and helped to recover under a heated lamp as well as given anti-inflammatory treatment of penicillin (Huabei Pharmaceutical Co., Ltd., Shijiazhuang, China) 8 × 105 U/d for 3 days. Electrocardiogram was performed on the third day after operation by 12-channel automatic analysis of electrocardiograph (FX-8322, FUKUDA DENSHI, Japan) and rats with six to eight lead Q waves in the standard 12-lead electrocardiogram were modeled successfully.

Finally, 96 rats with successful modeling and 20 rats underwent sham operation were included in this study. Model rats were randomly divided into four groups: model control group (*n* = 24), positive drug control group (*n* = 24), Buxinyishen powder (BXYS) lower dose group, BXYS (0.4 g/kg) group (*n* = 24) and BXYS higher dose group, BXYS (0.8 g/kg) group (*n* = 24). Drug was administered from the fourth day after surgery. Rats from sham group and model group were administered with carboxymethylcellulose sodium (Sigma, USA) according to their body weight (0.5 ml/100 g). Rats in BXYS groups were treated with 0.4 g/kg of BXYS in the lower dose group and 0.8 g/kg in the higher dose group twice a day. The rats in positive drug control group received fosinopril sodium (Sino American Shanghai Squibb Pharmaceutical, Shanghai, China) 4.67 mg/kg once a day. Both BXYS and fosinopril were administered with dissolution by carboxymethylcellulose sodium and the volume depended on body weight. After 28 days and 60 days of administration, rats were randomly selected from each group (n = 8) to perform following measurements.

#### Echocardiographic Assessment

Selected rats underwent intraperitoneal anesthesia by 1% pentobarbital sodium (45 mg/kg) and echocardiography were assessed (Vevo2100, VisualSonic, Canada) (**Supplementary Figure 2**). 2-D cine loops and guided M-mode frames were recorded from the parasternal short and long axis. All data were analyzed off-line with software resident on the ultrasound system. Left ventricular internal diameter at end-diastolic (LVIDd) and left ventricular internal diameter at end-systolic (LVIDs) were measured directly. Ejection fraction (EF) and fractional shortening (FS) were calculated automatically by the software.

#### Renal Hemodynamics

Selected rats underwent intraperitoneal anesthesia by 1% pentobarbital sodium (45 mg/kg) and ultrasound imaging system (Vevo2100, VisualSonic, Canada) was applied to assess the kidney hemodynamics (**Supplementary Figure 3**). The lateral position was taken to detect the peak systolic velocity (PSV) and end diastolic velocity (EDV) and resistance index (RI) of the intrarenal artery, including the main renal artery, segmental artery and interlobular artery. The blood supply of the kidneys in each group was evaluated by acceleration (AC).

#### Measurement of Neuroendocrine Factors

Blood samples were collected *via* abdominal aorta puncture using separation gel coagulation promoting tubes. Separation of serum was completed within half an hour after blood collection using low temperature high speed centrifuge (2-16PK, Sigma, Germany), at 4°C, 3000 g for 15 min. Serum samples were preserved into 1.5-ml cryotubes stored at −80°C in ultra-low temperature refrigerator (Thermo Scientific, USA) until analysis of each indicator within a short period of time. Serum concentrations of Ang II, cyclic adenosine monophosphate (cAMP), cyclic guanosine monophosphate (cGMP), C-reactive protein (CRP), brain natriuretic peptide (BNP), serum creatinine (Scr), and blood urea nitrogen (BUN) in plasma were determined by radioimmunoassay using a RIA kit (Beijing Kangyuan Ruide Biotechnology Co., Ltd., Beijing, China) following the manufacturer’s instructions.

#### The Distribution of CTGF in Serum, Urine and Renal Tissues

The selected rats in each group were kept in metabolic cages for 24 h to collect the urine samples. Renal tissues were stripped and frozen in liquid nitrogen immediately for further examinations. CTGF exists in its soluble form, sCTGF, in serum and urine. The sCTGF concentration in serum and urine samples were measured by commercial ELISA kits (CUSABIO, USA).

Tissue total RNA was extracted by TaKaRa MiniBEST Universal RNA Extraction Kit (Takara, Japan) and the concentration and integrity of CTGF mRNA gene were detected by agarose gel electrophoresis. The primers used were synthesized by Shanghai Shenggong Bioengineering Co., Ltd. The primer sequences were: CTGF (521 bp) upstream primer 5′-GAA AGA CAG GTA CTA GCT GA-3′, and the downstream primer 5′-CGTACC ATA TGT TCT GAC AG -3′; internal reference ACTIN (189 bp) upstream primer CCAAGAAGGAAGGCTGGAAAA. Other methods were performed with reference to the kit instructions, and amplification curves and dissolution profiles were performed by fluorescence quantitative PCR instrument (Applied Biosystems, USA). According to the detection results, the relative quantitative results of the target genes of each group were calculated according to the 2-^∆∆^ct relative quantitative calculation formula (Xu et al., 2016).

The immunohistochemistry (IHC) was used to analyze the expression of CTGF in rats’ kidney. Avidin-biotin-peroxidase complex commercial method (Beijing Boaosen Biotechnology Co., Ltd., Beijing, China) and the primary antibody (Anti-CTGF antibody, Abcam, USA) were used following the instructions. Pictures were analyzed by Image J software as described previously (Vrekoussis et al., 2009).

#### Histological Analysis

The harvested kidneys were fixed in 4% paraformaldehyde (InVitrogen, USA) for 48 h. After that, kidney tissues were embedded in paraffin and sectioned into 5 μm slices. Tissue sections were deparaffinized and stained with hematoxylin-eosin staining (HE, Biyuntian Biotechnology Co., Ltd., Shanghai, China) and Masson’s trichrome reagent (Thermo Scientific, Rockford, IL, USA). Quantitation of renal fibrosis was performed on cortical fields (at least 8 fields for each animal). Renal fibrosis was calculated based upon percentages of collagen positive areas in the total tissue area as described previously (Tang et al., 2015).

#### Determination of α-SMA and LRP Expression in Kidney Tissue

The experimental method is the same as CTGF. The primary antibody anti-alpha smooth muscle actin antibody and anti-low-density lipoprotein receptor-related protein 10 antibody were purchased from Abcam, USA.

### *In Vitro* Experiments

#### Cell Culture

NRK-52E cells were obtained from Peking Union Medical College Basic Medical Cell Center. Cells were cultured in DMEM supplemented (Gibco, USA) with 10% FBS (Gibco, USA) and penicillin streptomycin (100 IU/ml, Gibco, USA). Cell cultures were maintained at 37°C in a humidified incubator (Thermo Scientific, USA) containing 95% air and 5% CO_2_. Then cells were divided into the following five groups: BLANK group, BLANK + CTGF group, CHF group, CTGF + BXYS group and CHF + BXYS group. BLANK group was added serum of normal rats; BLANK + CTGF group was added serum of normal rats and exogenous CTGF (10 ng/ml); CTGF + BXYS group was added exogenous CTGF (10 ng/ml) and serum from normal rats which were treated with BXYS (0.8 g/kg) for seven days. CHF group was added serum of rats from model group and CHF + BXYS group was added serum of rats from BXYS (0.8 g/kg) group *in vivo* experiments. Serum from *in vivo* experiments rats was obtained after 60 days of administration. The detection was performed 48h after serum or CTGF added.

#### Reagents and Instruments

Chromatographic pure methanol (Fisher Scientific, USA); chromatographic purified water (DUKSAN, Korea); cell culture dish, cell culture bottle, 96-well cell culture plate (Corning, USA); ultra-clean workbench (Thermo Scientific, USA); pipetting gun (Eppendorf, Germany); inverted phase contrast microscope (OLYMPUS, Japan); microporous membrane (0.22 μm, Millipore, USA); microplate reader (BioTek, USA); Antifade Polyvinylpyrrolidone Mounting Medium (Biyuntian Biotechnology Co., Ltd., Shanghai, China); microporous membrane (0.22 μm, Millipore, USA); Western blot electrophoresis (Bio-Rad, USA); Western blot electric converter (Bio-Rad, USA); Decoloring shaker (3D, China); Vortex oscillator (SCILOGEX, USA).

#### Western Blot Analysis

Proteins were obtained from NRK-52E cells. Cells were homogenized in ice-cold lysis buffer (strong, Beijing ComWin Biotech Co., Ltd., Beijing, China) and then centrifuged at 12,000 g at 4°C for 20 min. NRK-52E cells were lysed with RIPA lysis buffer mixed with 1 µM phenylmethanesulfonyl fluoride (Biyuntian Biotechnology Co., Ltd., Shanghai, China) and 1 µM phosphatase inhibitor cocktail 3 (Sigma, USA). Cell protein concentrations were measured using a BCA Protein Assay Kit (Beijing ComWin Biotech Co., Ltd., Beijing, China). Protein samples were isolated by sodium dodecyl sulfate-polyacrylamide gel electrophoresis (Applygen, Beijing, China and then transferred to nitrocellulose membranes (Applygen, Beijing, China). After blocking with 5% non-fatmilk (Qxoid, England), the membranes were incubated at 4°C overnight with antibodies recognizing the following antigens: Anti-alpha smooth muscle Actin antibody (1:500–1,000 dilution, Abcam, USA) and GAPDH Rabbit Polyclonal Antibody (1:500–1,000 dilution, Applygen, China). After three washes with Tris-buffered saline-Tween (Sigma, USA), the membranes were incubated with IR Dye^®^-conjugated goat anti-rabbit (1:5,000 dilution, LI-COR Biosciences, Lincoln, NE, USA) for 1 h. The band intensities were scanned and quantified using an Odyssey infrared imaging system (Odyssey, LI-COP, USA). EasySee Western Marker (10–170 kDa) (Applygen, Beijing, China) was used to identify the molecular weight of proteins. Image J was used to quantitative analysis as described previously (Gallo-Oller et al., 2018). The final reported data of target protein were normalized by GAPDH.

#### Immunohistochemistry Analysis

IHC was used to analyze the expression ofα-SMA, LN, FN, Type I and III collagen in cells. The primary antibody as follows were used in the present study: Anti-alpha smooth muscle Actin antibody (Abcam, USA, ab5694), Anti-Low-density lipoprotein receptor-related protein 10 antibody (Abcam, USA, Ab111288), Anti-Collagen I antibody (Abcam, USA, Ab34710), Anti-Collagen III antibody (Abcam, USA, Ab7778), Anti-Fibronectin antibody (Abcam, USA, Ab2413), Anti-Laminin antibody (Abcam, USA, Ab128053). The secondary antibody labeled with fluorochrome diluted in 10% NGS (Alexa Fluor 488-labeled Goat Anti-Rabbit IgG (H+L) (Biyuntian Biotechnology Co., Ltd., Shanghai, China) was used. All of the images were obtained by an Eclipse TE2000-S electron fluorescence microscope (Nikon, Japan) and analyzed by Image J software as described previously (Vayrynen et al., 2012).

#### Statistical Analysis

Data analysis was performed using SPSS 20.0 (SPSS Inc., USA) and GraphPad Prism 7.0 (GraphPad Software, Inc., USA) software. Multiple comparisons were made by one-way analysis of variance tests. Data were presented as mean ± SD. A p-value < 0.05 was defined as statistically significant.

## Results

A CHF rat model induced by LAD ligation was used in this study. *In vitro* experiments, western blot analysis, real time polymerase chain reaction (RT-PCR) and other methods were used to clarify the role of CTGF in cardiorenal interactions from the whole tissue and cell level. All of the parameters were monitored 28 days and 60 days after drug treatment.

### *In Vivo* Experiments

#### BXYS Improved Heart Function in CHF Rats

**Figure 1:**
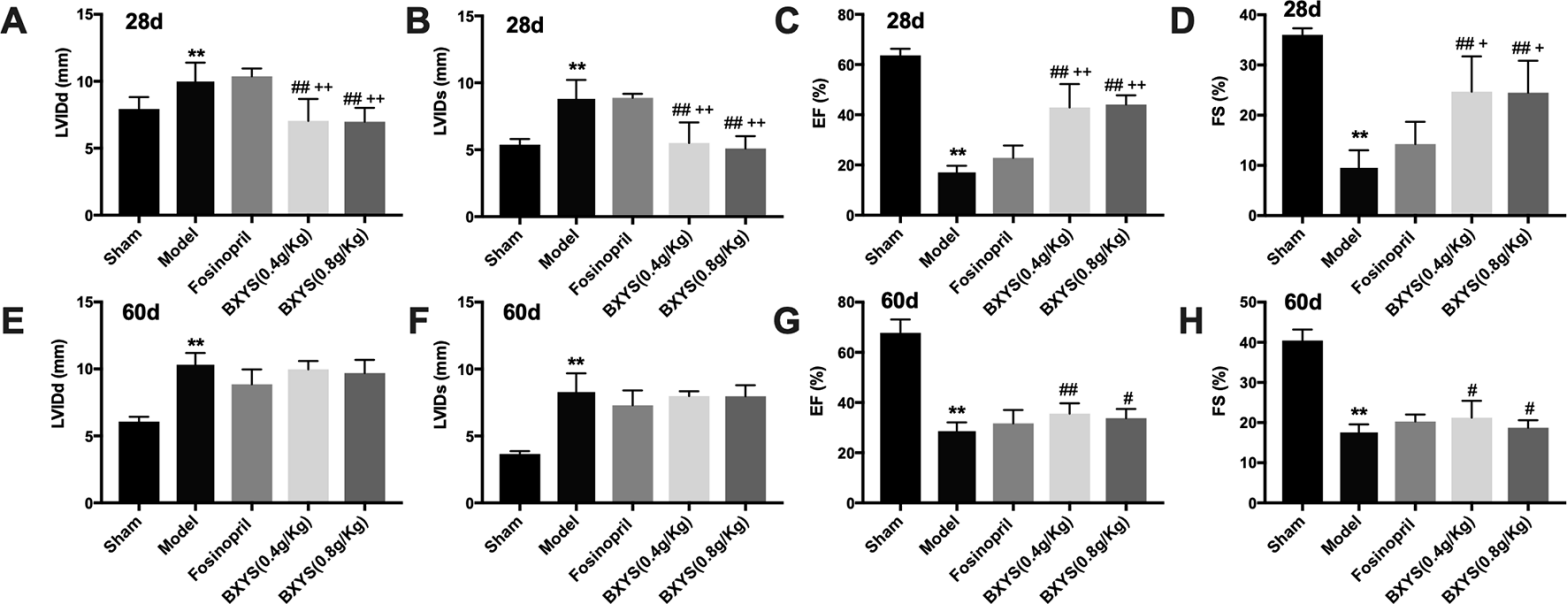
Echocardiographic assessment after 28 days and 60 days of drug administration. Sham, sham group; Model, model group; Fosinopril, fosinopril group; BXYS (0.4g/kg), Buxin Yishen 0.4g/kg treated group; BXYS (0.8g/kg), Buxin Yishen 0.8g/kg treated group. **P < 0.01 vs. sham group. ^#^P < 0.05, ^##^P < 0.01 vs. model group. ^+^P < 0.05, ^++^ P < 0.01 vs. fosinopril group. **(A)** Left ventricular internal diameter at end-diastolic (LVIDd) after 28 days. **(B)** Left ventricular internal diameter at end-systolic (LVIDs) after 28 days. **(C)** Ejection fraction (EF) after 28 days. **(D)** Fractional shortening (FS) after 28 days. **(E**–**H)** LVIDd, LVIDs, EF, FS after 60 days of drug administration.

Rats underwent echocardiographic evaluation 28 days and 60 days after drug treatment. After 28 days of administration, LVIDd and LVIDs in the model group were increased significantly (P < 0.01) (**Figures 1A, B**) with a marked reduction of EF and FS (P < 0.01) (**Figures 1C, D**) when compared with the sham group. Compared with the model group, all of these parameters were rescued in the two BXYS treatment groups (P < 0.01). Though fosinopril also showed positive effects on EF and FS, there were no statistical differences (P > 0.05). The comparison of two BXYS groups and fosinopril, as shown in **Figure 1**, demonstrated that BXYS had a better therapeutic effect on the heart function parameters LVIDd, LVIDs, EF and FS (P < 0.01). Similarly, after 60 days of administration, rats from the model group had higher LVIDd and LVIDs (P < 0.01) (**Figures 1E, F**) with lower EF and FS (P < 0.01) (**Figures 1G, H**) than those from the sham group. BXYS treatment groups, regardless of the dose, showed significant increase in EF and FS compared with the model group (P < 0.01, P < 0.05). Improvement of EF and FS was also found in the fosinopril group compared with those from the model group; however, the data were not statistically different (P > 0.05). There were no significant differences among the BXYS treatment groups and the fosinopril group after 60 days of administration (P > 0.05).

#### BXYS Treatment Improved Renal Hemodynamics

**Figure 2:**
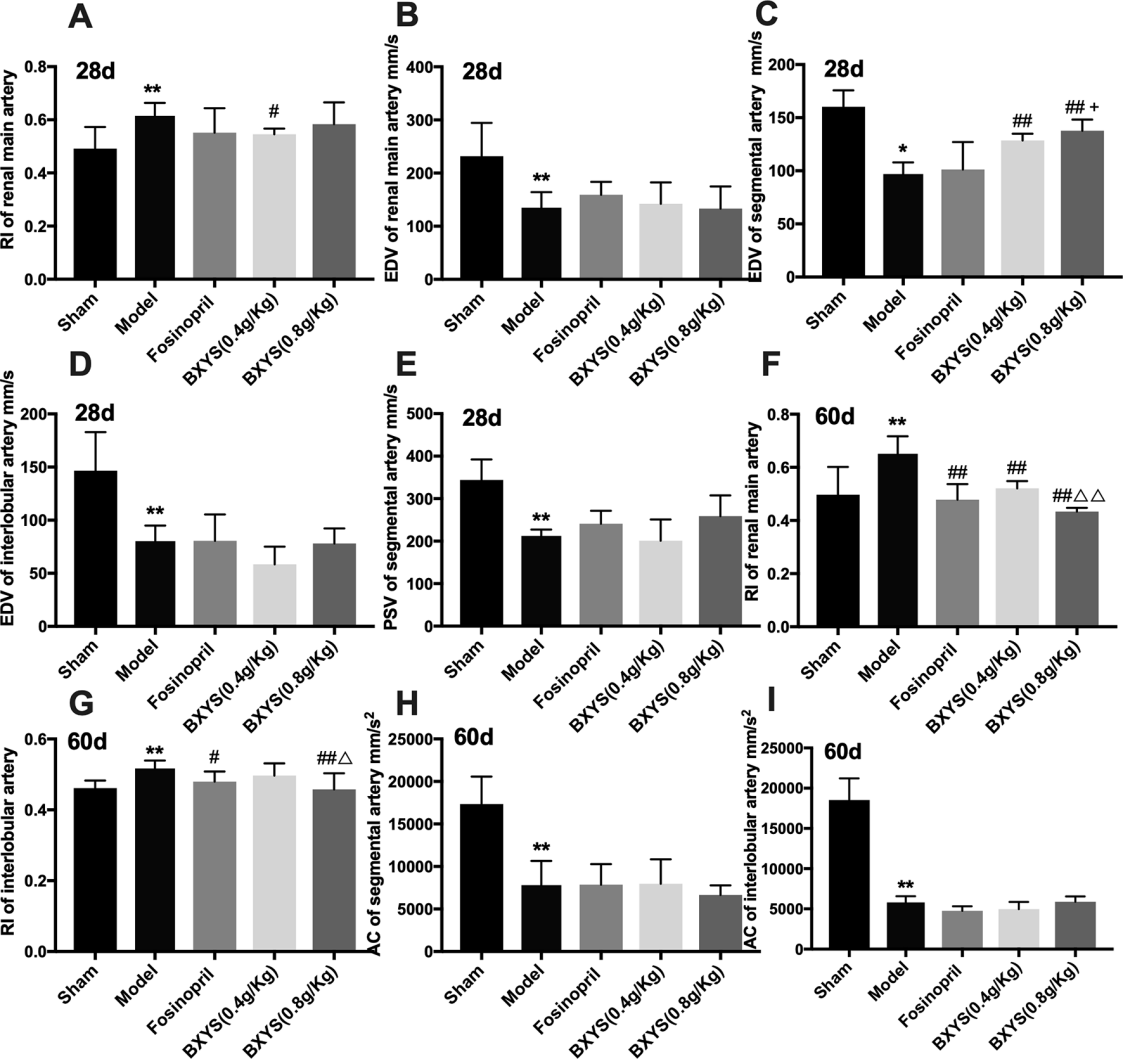
Renal hemodynamics after 28 days and 60 days of drug administration. Sham, sham group; Model, model group; Fosinopril, fosinopril group; BXYS (0.4g/kg), Buxin Yishen 0.4g/kg treated group; BXYS (0.8g/kg), Buxin Yishen 0.8g/kg treated group. *P < 0.05, **P < 0.01 vs. sham group. ^#^P < 0.05, ^##^P < 0.01 vs. model group. ^+^P < 0.05 vs. fosinopril group. ^Δ^P < 0.05, ^ΔΔ^P < 0.01 vs. BXYS (0.4g/kg) group. **(A)** Renal main artery resistance index (RI) after 28 days. **(B)** Renal main artery end diastolic velocity (EDV) after 28 days. **(C)** Segmental artery EDV after 28 days. **(D)** Interlobular artery EDV after 28 days. **(E)** Segmental artery peak systolic velocity (PSV) after 28 days. **(F)** Renal main artery RI after 60 days. **(G)** Interlobular artery RI after 60 days. (**H)** Segmental artery acceleration (AC) after 60 days. **(I)** Interlobular artery AC after 60 days.

To evaluate kidney dysfunction induced by CHF, PSV, EDV, RI, and AC of main renal artery, segmental artery and interlobular artery were detected. After 28 days of administration, model rats showed higher renal main artery RI (P < 0.05) (**Figure 2A**); lower renal main artery, segmental artery and interlobular artery EDV (P < 0.05, P < 0.01) (**Figures 2B–D**); and lower segmental artery PSV (P < 0.01) (**Figure 2E**) compared with the sham group. Treatment with both 0.4 g/kg and 0.8 g/kg of BXYS improved segmental artery EDV (P < 0.01) compared with the model group. Only the middle-dose of BXYS was correlated with a significant change in main renal artery RI (P < 0.05). Furthermore, higher-dose treatment of BXYS was found to be superior to fosinopril in improving segmental artery EDV (P < 0.05). Other parameters had no significant differences among the groups. After 60 days of administration, renal main artery and interlobular artery RI increased significantly (P < 0.01) (**Figures 2F, G**) with a marked decrease segmental and interlobular artery AC (P < 0.01) (**Figures 2H, I**) in the CHF model compared with sham group. Fosinopril, BXYS (0.4 g/kg) and BXYS (0.8 g/kg) groups showed improvement in renal main artery RI compared with the model group (P < 0.01). However, the improvement of interlobular artery RI was also found in rats from the fosinopril and BXYS (0.8 g/kg) groups (P < 0.01, P < 0.05) but not the BXYS (0.4 g/kg) group. Compared with the lower dose BXYS treatment, higher dose treatment more effectively rescued renal main artery and interlobular artery RI (P < 0.01).

#### BXYS Showed Positive Effect on Neuroendocrine Factors

**Figure 3:**
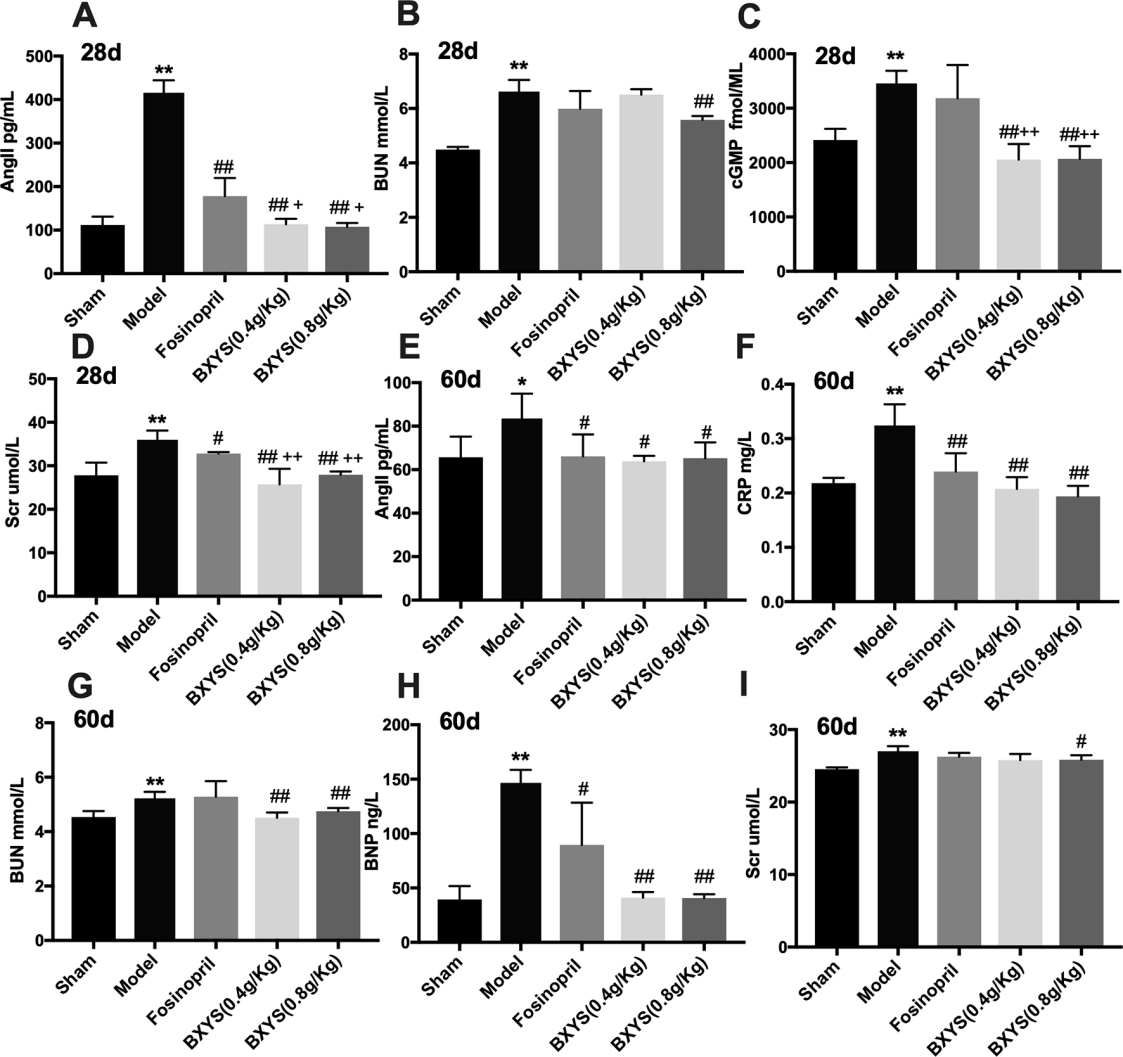
Measurement of neuroendocrine factors after 28 days and 60 days of drug administration. Sham, sham group; Model, model group; Fosinopril, fosinopril group; BXYS (0.4g/kg), Buxin Yishen 0.4 g/kg treated group; BXYS (0.8 g/kg), Buxin Yishen 0.8 g/kg treated group. *P < 0.05, **P < 0.01 vs. sham group. ^#^P < 0.05, ^##^P < 0.01 vs. model group. ^+^P < 0.05, ^++^P < 0.01 vs. fosinopril group. **(A)** Serum concentration of angiotensin II (Ang II) after 28 days. **(B)** Serum concentration of blood urea nitrogen (BUN) after 28 days. **(C)** Serum concentration of cyclic guanosine monophosphate (cGMP) after 28 days. **(D)** Serum creatinine (Scr) after 28 days. **(E)** Serum concentration of Ang II after 60 days. **(F)** Serum concentration of C reaction protein (CRP) after 60 days. **(G)** Serum concentration of BUN after 60 days. **(H)** Serum concentration of brain natriuretic peptide (BNP) after 60 days. **(I)** Serum concentration of Scr after 60 days.

After 28 days of administration, compared with the sham group, serum samples from the model group showed higher concentrations of AngII, BUN, cGMP, and Scr (P < 0.01) (**Figures 3A–D**). These four parameters were reduced by higher dose treatment of BXYS (P < 0.01) when compared with model group. Lower dose BXYS and fosinopril lowered serum concentrations of AngII, Scr, cGMP (P < 0.01, P < 0.05) but failed to lower BUN (P > 0.05). Compared with fosinopril, both two BXYS treatment groups showed lower serum concentrations of AngII, cGMP, and Scr. Other neuroendocrine factors including cAMP, BNP, and CRP had no statistical differences among groups (P > 0.05). After 60 days of administration, serum concentrations of AngII, CRP, BUN, BNP, and Scr were higher in the model rats compared with those from the sham group (P < 0.01, P < 0.05) (**Figures 3E–I**). These factors were down-regulated by 0.8 g/kg of BXYS treatment (P < 0.05, P < 0.01) compared with the model. 0.4 g/kg of BXYS administration showed significant reduction of CRP, BNP, AngII and BUN (P < 0.05, P < 0.01) but had a limited positive effect on Scr (P > 0.05). Fosinopril lowered serum concentrations of AngII, CRP, BNP compared with the model (P < 0.05, P < 0.01). We also found that compared with fosinopril, higher dose of BXYS might have a better effect on decreasing concentrations of CRP and BNP, but there were no statistical differences (P > 0.05). Other indicators such as cAMP and cGMP showed no significant differences among groups (P > 0.05).

#### BXYS Down-Regulated the Expression of CTGF

**Figure 4:**
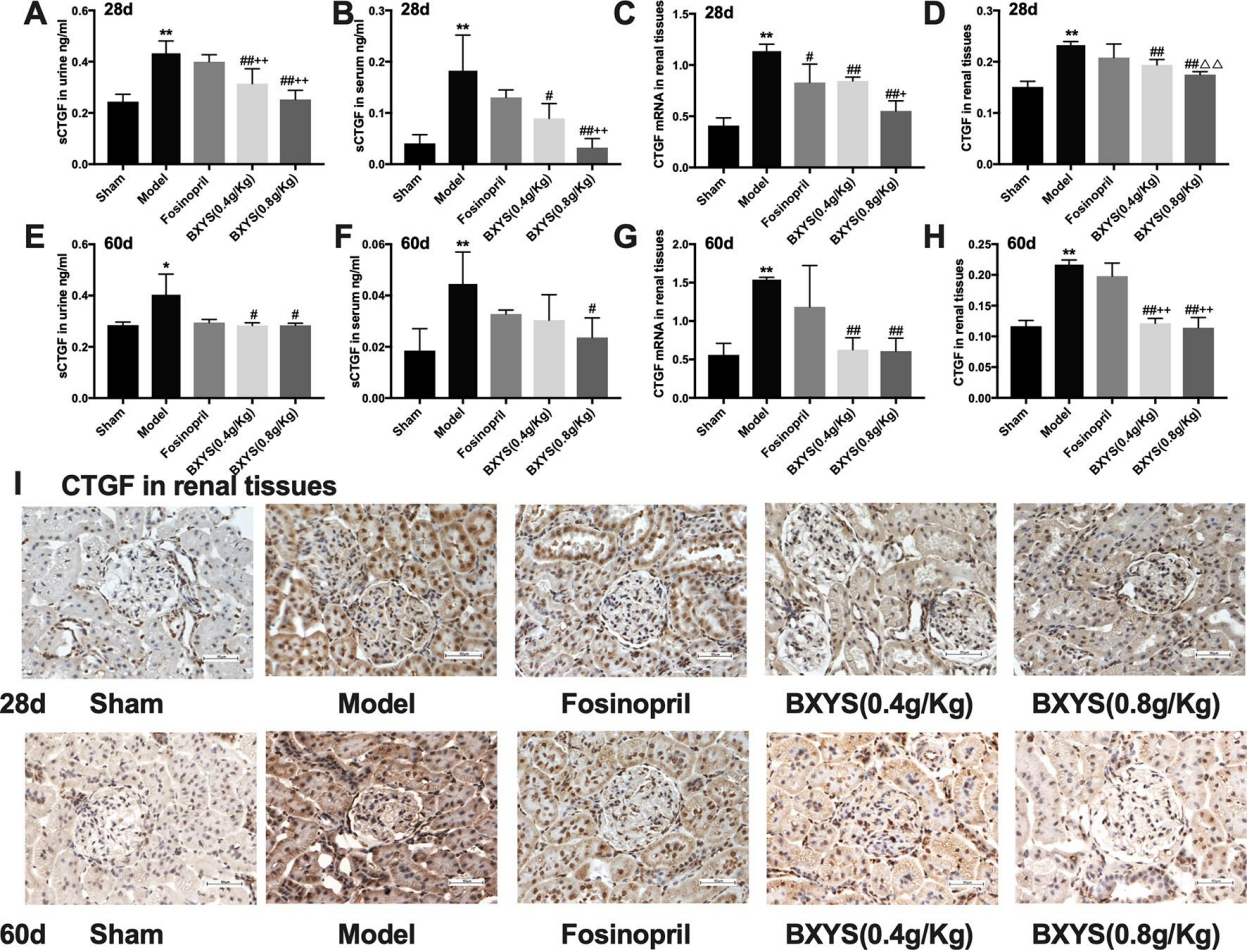
sCTGF concentration in serum, urine and CTGF mRNA, CTGF expression in renal tissues after 28 days and 60 days of drug administration. Sham, sham group; Model, model group; Fosinopril, fosinopril group; BXYS (0.4 g/kg), Buxin Yishen 0.4 g/kg treated group; BXYS (0.8 g/kg), Buxin Yishen 0.8g/kg treated group. *P < 0.05, **P < 0.01 vs. sham group. ^#^P < 0.05, ^##^P < 0.01 vs. model group. ^+^P < 0.05, ^++^P < 0.01 vs. fosinopril group. ^△△^P< 0.01 vs. BXYS (0.4 g/Kg) group. **(A)** Soluble connective tissue growth factor (sCTGF) levels in urine after 28 days. **(B)** sCTGF levels in serum after 28 days. **(C)** CTGF mRNA expression in renal tissues after 28 days. **(D)** CTGF expression in renal tissues after 28 days. **(E)** sCTGF levels in urine after 60 days. **(F)** sCTGF levels in serum after 60 days. **(G)** CTGF mRNA expression in renal tissues after 60 days. **(H)** CTGF expression in renal tissues after 60 days. **(I)** CTGF expression in renal tissues (×400 magnification). Upper panel shows analysis after 28 days of administration and the lower panel shows analysis after 60 days of administration.

CTGF exists in its soluble form, sCTGF, in serum and urine. Both two time points of measurement showed higher serum and urine sCTGF concentration in the model group compared with the sham group (P < 0.01, P < 0.05) (**Figures 4A, B, E, F**). After 28 days of administration, two BXYS treatment groups showed lower serum and urine sCTGF concentration (P < 0.01, P < 0.05) compared with the model. However, fosinopril did not have a similar effect on serum or urine sCTGF concentration (P > 0.05). After 60 days of administration, only BXYS (0.8 g/kg) group significantly decreased serum and urine sCTGF concentration (P < 0.05) compared with the model. BXYS (0.4 g/kg) decreased urine sCTGF concentration (P < 0.05), but serum concentration was not significantly decreased (P > 0.05). Similarly to the 28-day treatment results, fosinopril showed no decreasing effect on CTGF expression (P > 0.05).

CTGF mRNA and CTGF protein in renal tissues were also measured to identify the distribution of CTGF. At two time points of measurement, CTGF mRNA expression levels were higher in the model group than the sham group (P < 0.01) (**Figures 4C, G**), but both two dosage of BXYS decreased CTGF mRNA expression (P < 0.01). Fosinopril treatment reduced CTGF mRNA expression at 28 days after drug administration (P < 0.05), but a similar effect was not observed after 60 days of treatment (P > 0.05). Furthermore, compared with the fosinopril group, BXYS (0.8 g/kg) group showed lower CTGF mRNA expression (P < 0.05) after 28 days of treatment, but this effect was not observed after 60 days of treatment (P > 0.05). CTGF protein expression showed a similar tendency as CTGF mRNA. As showed in **Figure 4I**, CTGF brown granules were observed in cytoplasm of the mesangial cells, renal tubular epithelial cells and interstitial cells in model group. Three drug treated groups showed different degrees of reduction in CTGF granules. Quantitative analysis showed that CTGF protein was significantly up-regulated in the model group compared with the sham group after 28 days and 60 days of administration (P < 0.01) (**Figures 4D, H**). Both two BXYS treatment groups showed lower expression of CTGF than the model group at these two time points (P < 0.01). In addition, higher dose of BXYS had lower CTGF expression than lower dose after 28 days of treatment (P < 0.01). Fosinopril also had a decreasing effect on CTGF expression, but there were no statistical differences (P > 0.05).

#### BXYS Protected Renal Tissue Structure and Decreased the ECM Deposition

**Figure 5:**
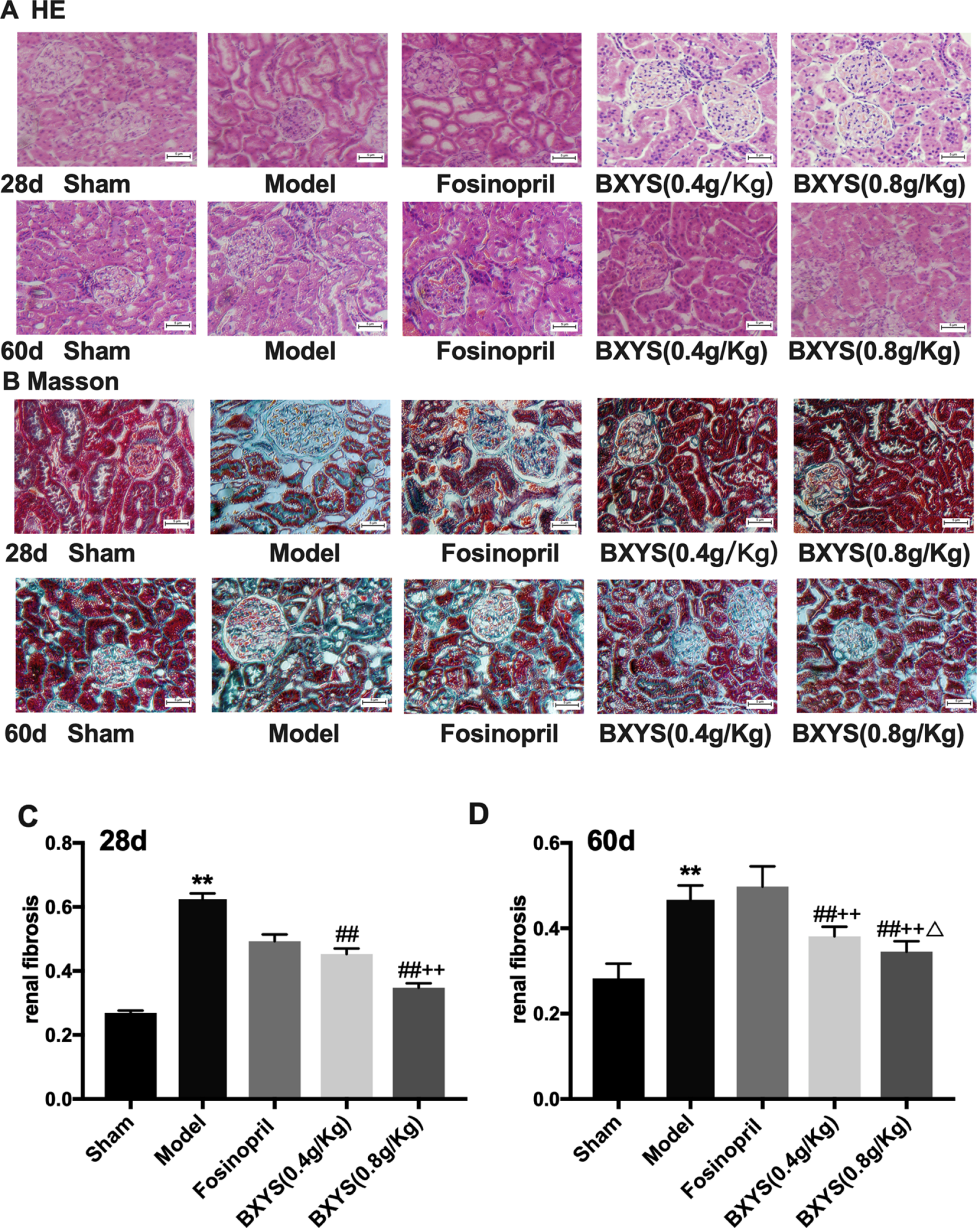
Histological analysis after 28 days and 60 days of drug administration. Sham, sham group; Model, model group; Fosinopril, fosinopril group; BXYS (0.4 g/kg), Buxin Yishen 0.4 g/kg treated group; BXYS (0.8 g/kg), Buxin Yishen 0.8 g/kg treated group. **P < 0.01 vs. sham group. ^##^P < 0.01 vs. model group. ^++^P < 0.01 vs. fosinopril group. ^Δ^P < 0.05 vs. BXYS (0.4g/kg) group. **(A)** Tissue sections were deparaffinized and stained with hematoxylin-eosin staining (HE) (×200 magnification). **(B)** Tissue sections were stained by Masson’s trichrome reagent (×200 magnification). **(C** and **D)** Quantitation of renal fibrosis after 28 days and 60 days.

HE staining of kidney tissue showed normal renal tissue structure in the sham group (**Figure 5A**). There were no pathological changes in glomerular, renal tubules and renal interstitial. At 28 days after administration, compared with the sham group, renal tubules of the model group were significantly dilated, and there was focal infiltration by a large number of interstitial inflammatory cells. The degree of tubular dilatation was reduced in the fosinopril, BXYS (0.4 g/kg) and BXYS (0.8 g/kg) groups compared with the model group, with only scattered infiltration by interstitial cells. At 60 days after administration, renal tubules of the model rats were significantly dilated, and the infiltration of interstitial inflammatory cells was increased. Compared with the model group, tubular dilatation and interstitial inflammatory cell infiltration were alleviated and significantly reduced, respectively, in the three drug treatment groups.

Masson’s Trichrome staining showed normal structure with a small amount of distributed ECM in the sham group, (**Figure 5B**). However, a large amount of ECM and fibrosis were observed in the model group. The fosinopril group showed partial ECM. Two BXYS treatment groups showed a small amount of ECM in renal tissue. Quantitative analysis was also performed. Both 28-day and 60-day measurements showed a significant increase of ECM in the model group compared with the sham group (P < 0.01) (**Figures 5C, D**). Two BXYS treatment groups showed less amount of ECM than the model group (P < 0.01). However, fosinopril treatment failed to reduce ECM at two time points (P > 0.05). It is worth mentioning that high-dose BXYS decreased ECM significantly compared with middle-dose at 60 days after drug treatment (P < 0.05).

#### BXYS Treatment Decreased α-SMA and LRP Expression in Kidney Tissue

**Figure 6:**
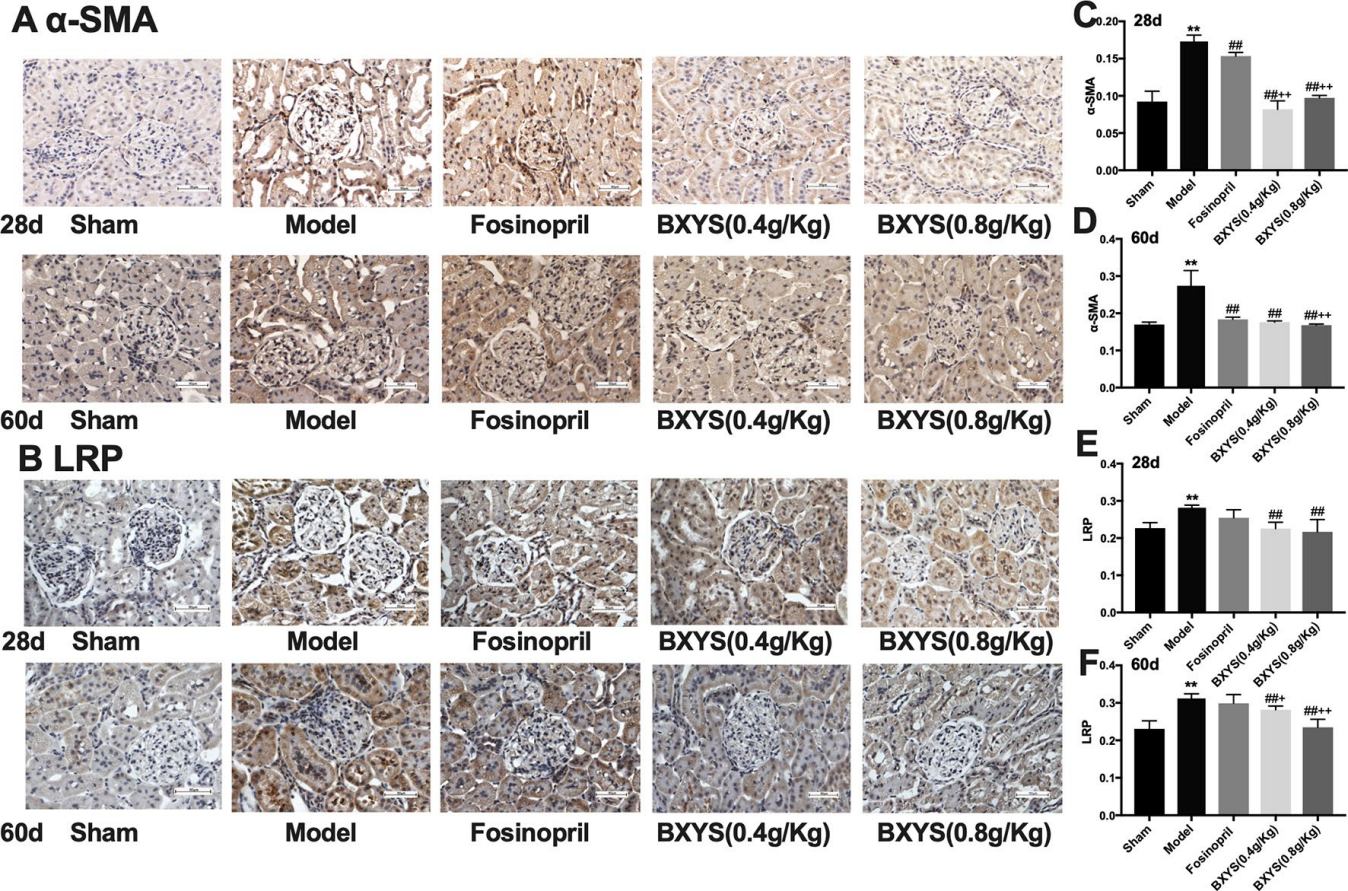
Determination of α-SMA and LRP expression in kidney tissue after 28 days and 60 days of drug administration. Sham, sham group; Model, model group; Fosinopril, fosinopril group; BXYS (0.4 g/kg), Buxin Yishen 0.4 g/kg treated group; BXYS (0.8 g/kg), Buxin Yishen 0.8 g/kg treated group. **P <0.01 vs. sham group. ^##^P < 0.01 vs. model group. ^+^P < 0.05, ^++^P < 0.01 vs. fosinopril group. **(A)** Alpha smooth muscle Actin (牀α*-SMA)* expression in kidney tissue (×400 magnification). **(B)** Low density lipoprotein receptor-related protein (LRP) expression in kidney tissue (×400 magnification). **(C** and **D)** Quantitative analysis of α*-SMA* expression after 28 days and 60 days. **(E** and **F)** Quantitative analysis of LRP expression after 28 days and 60 days.

As showed in **Figures 6A, B**, α-SMA and LRP brown granules were observed in cytoplasm of the mesangial cells, renal tubular epithelial cells and interstitial cells in model group. Three drug treated groups showed different degrees of reduction in α-SMA and LRP granules at 28d and 60d. Quantitative analysis showed that after 28 days of administration, the expressions of α-SMA and LPR were higher in the model group compared with the sham group (P < 0.01) (**Figures 6C, E**). Compared with the model group, all three drug treatment groups showed a significant reduction in α-SMA and LPR expression (P < 0.01). Both two BXYS treatment groups showed lower α-SMA expression compared with the fosinopril group (P < 0.01), but this effect was not observed for LRP expression (P > 0.05). After 60 days of administration, α-SMA and LRP still maintained higher expression in the model group compared with the sham group (P < 0.01) (**Figures 6D, F**). Similarly, two BXYS groups decreased α-SMA and LRP expression significantly compared with the model group (P < 0.01). Fosinopril also lowered α-SMA expression significantly (P < 0.01) but not LRP expression (P > 0.05). Compared with fosinopril, higher dose BXYS was more effective at decreasing both LRP and α-SMA expression (P < 0.01).

### *In Vitro* Experiments

#### BXYS Decreased α-SMA and LRP Expression in Cells

**Figure 7:**
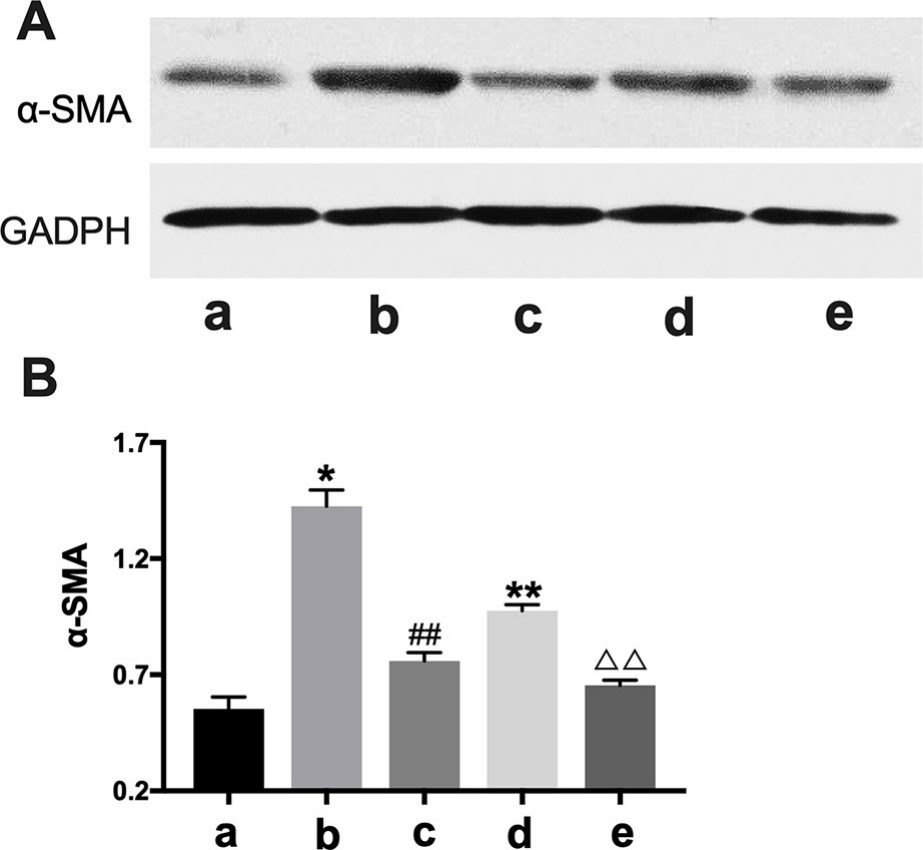
Western blot analysis of α*-SMA* expression. CTGF, connective tissue growth factor. BXYS, Buxin Yishen decoction. CHF, chronic heart failure. α*-SMA*: alpha smooth muscle actin. a: BLANK group. b: BLANK+CTGF group. c: CTGF+BXYS group. d: CHF group. e: CHF+BXYS group. *P < 0.05, **P < 0.01 vs. BLANK group. ^##^P < 0.01 vs. BLANK+CTGF group. ^ΔΔ^P < 0.01 vs. CHF group. **(A)** α-SMA expression in cells. **(B)** Quantitative analysis of western blot.

In western blot analysis, the BLANK+CTGF group and the CHF group showed higher α-SMA expression compared with BLANK group (P < 0.05, P < 0.01) (**Figure 7**), indicating that α-SMA was up-regulated by CTGF, and that the CHF group might have the same biomarker. After treatment with BXYS, α-SMA levels were decreased as shown in the CTGF+BXYS group and the CHF+BXYS group (P < 0.01).

**Figure 8:**
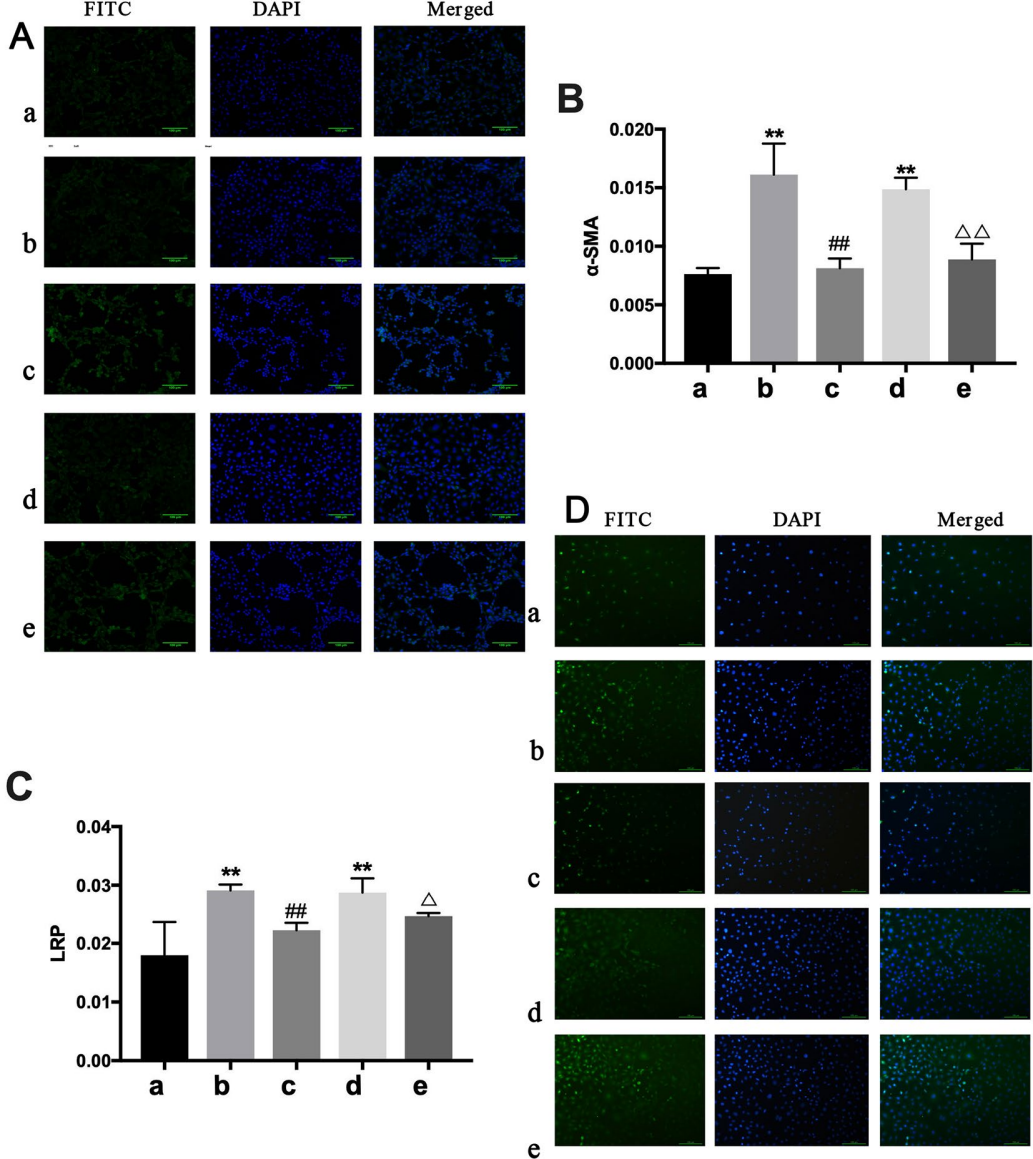
Determination of α*-SMA* and LRP in NREK-52E cells. FITC, fluorescein isothiocyanate. DAPI, 4′,6-diamidino-2-phenylindole. CTGF, connective tissue growth factor. BXYS, Buxin Yishen decoction. CHF, chronic heart failure. a: BLANK group. b: BLANK+CTGF group. c: CTGF+BXYS group. d: CHF group. e: CHF+BXYS group. **P < 0.01 vs. BLANK group. ^##^P < 0.01 vs. BLANK+CTGF group. ^Δ^P < 0.05, ^ΔΔ^P < 0.01 vs. CHF group. **(A** and **B)** Alpha smooth muscle actin (α-SMA) expression in cells. **(C** and **D)** Low density lipoprotein receptor-related protein (LRP) expression in cells.

IHC analysis showed only α-SMA and LPR had significant expression differences among groups. Α-SMA and LPR expression were higher in the BLANK+CTGF group and the CHF group compared with the BLANK group (P < 0.01) (**Figures 8B, C**). Treated with BXYS, α-SMA and LRP expression was decreased significantly (P < 0.01, P < 0.05). Results of cellular immunofluorescence were similar upon quantitative analysis (**Figures 8A, D**).

## Discussions

Previous studies have shown that renal dysfunction is prevalent in people with CHF and is an independent negative, prognostic factor for CHF (McAlister et al., 2004). Accumulating evidence suggests that three major mechanisms contribute to the progression of cardiorenal interactions, which include hemodynamic, neurohormonal, cardiovascular disease-associated mechanisms. All three mechanisms can negatively affect both cardiac and renal function (Schefold et al., 2016). In patients with type 2 CRS, hemodynamic changes induced by CHF cause the activation of renin-angiotensin-aldosterone system and release neurotransmitters including nitric oxide (NO), BNP and AngII, which can subsequently lead to renal vasoconstriction, hypoxia, cytokine release, inflammation, and renal fibrosis (Jois and Mebazaa, 2012). Fosinopril belongs to the third generation of long-acting ACEI drugs. By inhibiting the activity of angiotensin converting enzyme, fosinopril blocks the conversion of angiotensin I to AngII (Prasad et al., 2004). It has previously been observed that fosinopril can reduce AngII serum concentration, increase left ventricular EF, and improve heart systolic function in patients with CHF (Ma et al., 2009). A study by Liu YM (Liu et al., 2010) reported that fosinopril decreased the expression of CTGF and delayed the development of renal fibrosis in rats. Therefore, fosinopril was chosen as a positive control to observe the effect of BXYS on heart function and the process of renal fibrosis development. Moreover, our previous studies (Qiu et al., 2010) showed obvious cardiac function decline at 28d and 60d in model rats, so we chose these two detection points to observe the develop of CRS and to explore differences in the therapeutic effects of BXYS.

BNP is secreted mainly by the ventricles and is a sensitive and specific indicator of ventricular dysfunction, which has become an important biomarker of cardiac function in CHF patients (Celik et al., 2012; Feldman et al., 2013). It has been widely recognized that RASS and AngII play critical roles in the development of renal fibrosis (Ruster and Wolf, 2011; Chang et al., 2016) and in which CTGF is the key factor (Gu et al., 2012; Montford and Furgeson, 2017). As a downstream factor of transforming growth factor-β1, CTGF might have an independent biological role in the development of organ fibrosis, evidenced by CTGF expression correlation to CRP and BNP serum concentrations (Rosin et al., 2013). LRP is a cell surface receptor recognized by several growth factors, including CTGF (Mason, 2013; Wujak et al., 2017), that maintains the integrity of blood vessels, partially by modulating CTGF expression (Muratoglu et al., 2013). The α-SMA, an actin isoform, plays an important role in fibrogenesis (Zeniya et al., 2017) and can reflect the degree of renal fibrosis (Lu et al., 2018).

Type 2 CRS is characterized by chronic cardiac dysfunction that causes progressive chronic kidney disease. BXYS formula is a Chinese clinical formula consisting of six herbal species. Studies of Zhu H et al. (Zhu et al., 2008) found that *Astragali Radix* (*Astragalus membranaceus* (Fisch.) Bunge var. *mongholicus* (Bunge) Hsiao) extract could alleviate myocardial reperfusion injury. *Aconiti Lateralis Radix Praeparata* (*Aconitum carmichaeli* Debx.) had been reported to have potent anti-inflammatory properties (Murayama et al., 1991) and is commonly used in the treatment of CHF (Fu et al., 2010). *Salviae Miltiorrhizae Radix Et Rhizoma* (*Salvia miltiorrhiza* Bunge) promotes blood circulation and has cardioprotective effects (Chang et al., 2006). *Poria* [*Poria cocos* (Schw.) Wolf] is commonly used to patients with renal edema for its diuretic effect (He et al., 2019). In traditional Chinese medicine theory, the combination of these medicines showed positive effectiveness in improving heart function, promoting blood circulation and alleviating water retention at the same time by intercourse between heart and kidney. Further more, it was characteristic for traditional Chinese medicines to have a higher therapeutic dose than those with single ingredient. Taking marketed drugs as examples, Ganmao Qingre Keli (Beijing Tongrentang Pharmacy Co., Ltd.) is recommended 12 g twice a day in clinic for adults, and Niuhuang Qingxin Wan (Beijing Tongrentang Pharmacy Co., Ltd.) is used 3-6 g twice a day for adults. In the present study, rats were treated twice a day at 0.4 g/kg (equivalent to 1× human recommended dose, about 3.87 g/60 kg) or 0.8 g/kg (equivalent to 2× human recommended dose, about 7.74 g/60 kg). Both two doses were well tolerated in these rats.

The present study showed that BXYS treatment had impressive effects on cardiac function and renal hemodynamics in CHF rats. Compared with fosinopril, BXYS showed superiority in many aspects including improving heart function and renal hemodynamics, improving renal blood biochemical parameters, decreasing ECM deposition, and delaying the progression of type 2 CRS in the model group. Compared with 0.4 g/kg of BXYS, 0.8 g/kg of BXYS had better effects in this study. What’s more, BXYS had different therapeutical effects as the disease developed. The most obvious difference was that at 28d after drug administration, BXYS rescued LVIDd, LVIDs, EF and FS significantly. At 60d, BXYS still improved EF and FS obviously, and had a tendency to improve LVIDd and LVIDs though there were no statistical differences. It demonstrated that BXYS could delay myocardial remodeling with the ventricular enlargement as the main manifestation during the early progress of heart failure. While in the later stage of heart failure, BXYS could hardly reverse ventricular hypertrophy. Therefore, in clinical practice, BXYS may have a better effect on the early stage of heart failure patients. For the ACEI drug, fosinopril showed positive effects in renal hemodynamics improvement and neuroendocrine factors decrease, such as AngII and CRP, but no significant improvement of heart function was observed in the present study although it had been reported to prevent left ventricular remodeling (Borghi et al., 1998). These results were consisting to our previous study (Qiu et al., 2018).

**Figure 9:**
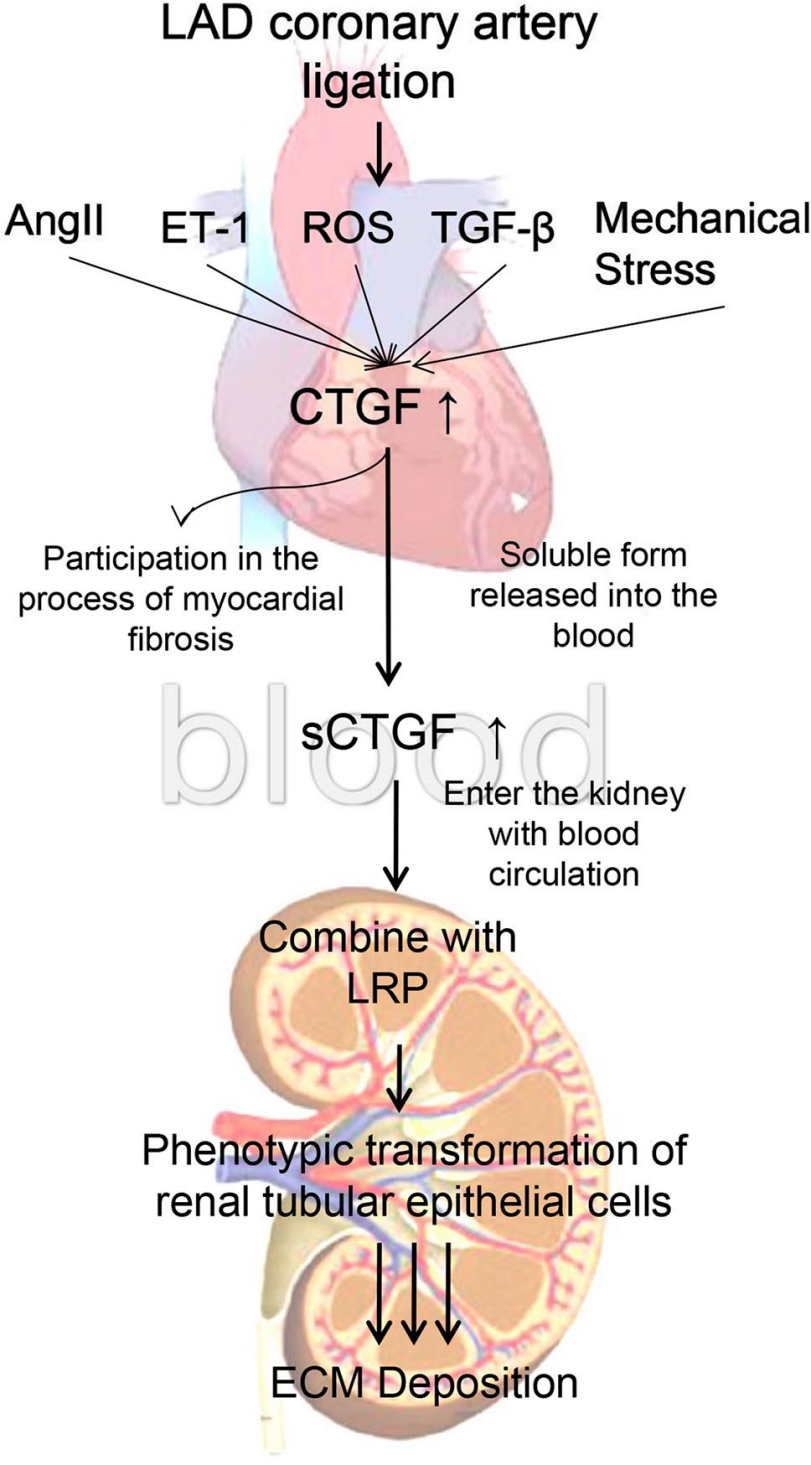
Mechanism of cardiac-renal connection. LAD, left anterior descending. AngII, angiotensin II. ET-1, endothelin 1. ROS, reactive oxygen species. TGF-β, transforming growth factor-β. CTGF, connective tissue growth factor. sCTGF, soluble form CTGF. LRP, low-density lipoprotein receptor-related protein. ECM, extracellular matrix.

*In vitro* experiments, since the 0.8 g/kg of BXYS had better effect on relaying renal fibrosis than 0.4 g/kg *in vivo* study, we chose 0.8 g/kg of BXYS treatment *in vitro* study. CHF group showed higher expression in LRP and α-SMA which was similar to induction by CTGF stimulation in BLANK+CTGF group. The mechanism by which BXYS works may be that it reduces the expression of CTGF and its receptor, LRP. This reduction might be related to the phenotypic transformation of cells, which delays the process of renal fibrosis induced by CHF (Matsui and Sadoshima, 2004; Mason, 2013) (**Figure 9**). It indicated that the CTGF-LRP pathway might be the target of BXYS for prevention and treatment of type 2 CRS caused by myocardial infarction. However, serum components of CHF animals are complex. Besides CTGF, there may be other cytokines inducing these changes. Further research is needed.

The study had several limitations as well. First, the mechanism by which the cardiac-renal connection occurs in myocardial infarction caused type 2 CRS remains unclear, although we conducted the pilot study and found the potential target CTGF-LRP. Further exploration about this target is worthy conducted. Second, the BXYS preparation technology needs be optimized to reduce the daily administrated dose which might associate the improvement of tolerance and compliance.

## Conclusion

The current study provided evidences that BXYS played a role in improving heart function and delaying the progress of renal fibrosis, meanwhile CTGF-LRP pathway showed a positive change after treated with BXYS, which might be a new target and therapeutic strategy for myocardial infarction caused type 2 CRS and is worthy of further exploration.

## Data Availability Statement

The raw data supporting the conclusions of this manuscript will be made available by the authors, without undue reservation, to any qualified researcher.

## Ethics Statement

All the animal experiments were performed in accordance with the China Physiological Society’s “Guiding Principles in the Care and Use of Animals” with the approval of the Animal Care Committee of Beijing Capital Medical University (license No. AEEI-2016-180).

## Author Contributions

QQ wrote the manuscript. QQ and JC performed experiments *in vivo* and *in vitro*. YuW contributed the renal hemodynamics analysis. XH performed the cardiac function analysis. QQ, YoW, YZ, and YM edited the manuscript. YL guided the research. All authors read and approved the final manuscript.

## Funding

This work was supported by National Natural Science Foundation of China (No.81403200; No.81822049) and National Major Scientific and Technological Special Project for “Significant New Drugs Development” during the Thirteenth Five-year Plan Period (No. 2017ZX09304017)

## Conflict of Interest

The authors declare that the research was conducted in the absence of any commercial or financial relationships that could be construed as a potential conflict of interest.

The handling editor declared a shared affiliation, though no other collaboration with several of the authors, QQ, JC, YoW, YZ, YuW, XH, YM, and YL at the time of the review.
