# Supplementary figures and images for: Time Course of the Effects of Buxin Yishen Decoction in Promoting Heart Function and Inhibiting the Progression of Renal Fibrosis in Myocardial Infarction Caused Type 2 Cardiorenal Syndrome Rats
Source: Front Pharmacol. 2019 Oct 23;10:1267. doi: 10.3389/fphar.2019.01267 (PMC6819435; doi:10.3389/fphar.2019.01267)

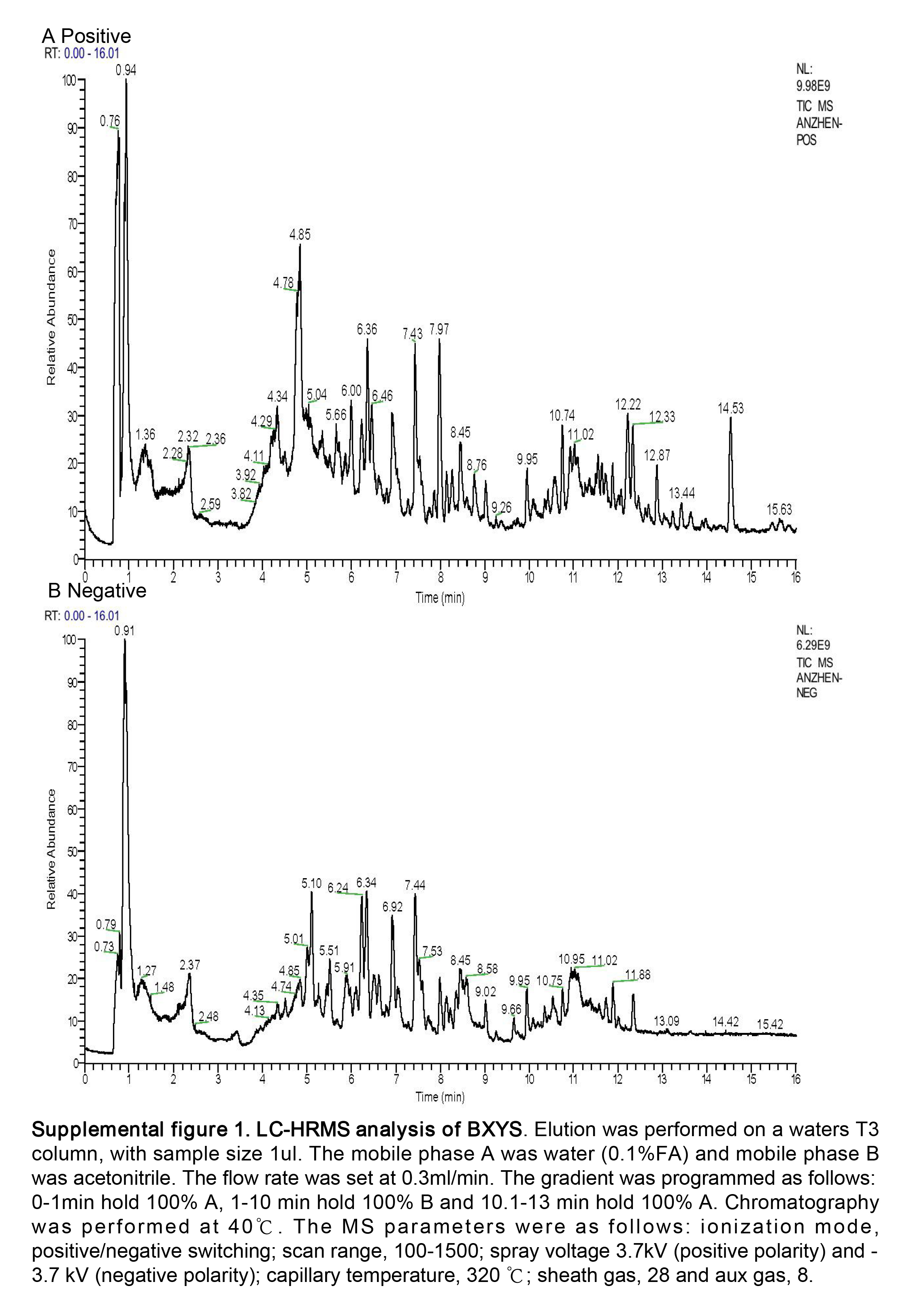

Supplement: Supplementary file 1 [file Image_1.jpeg]

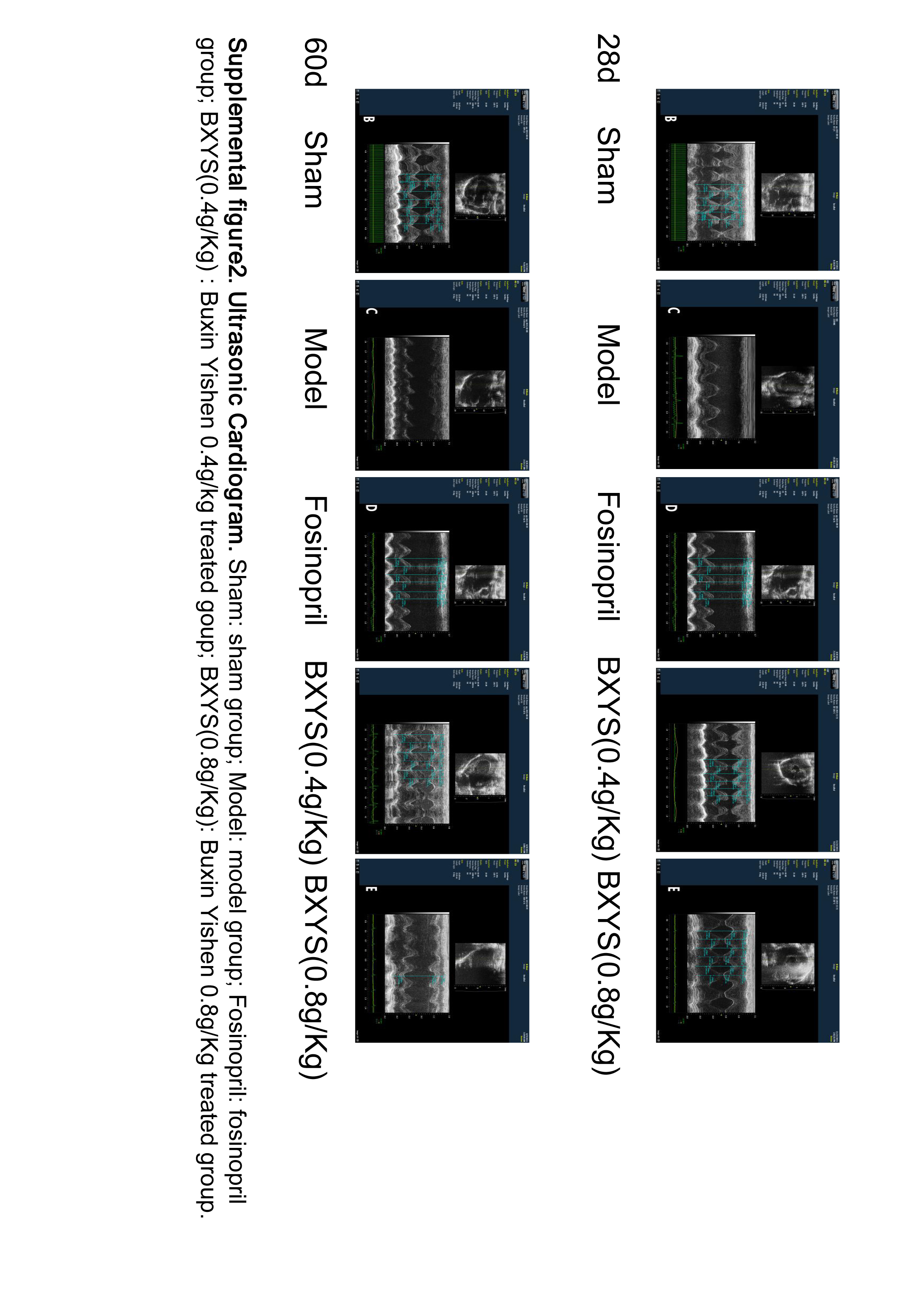

Supplement: Supplementary file 2 [file Image_2.jpeg]

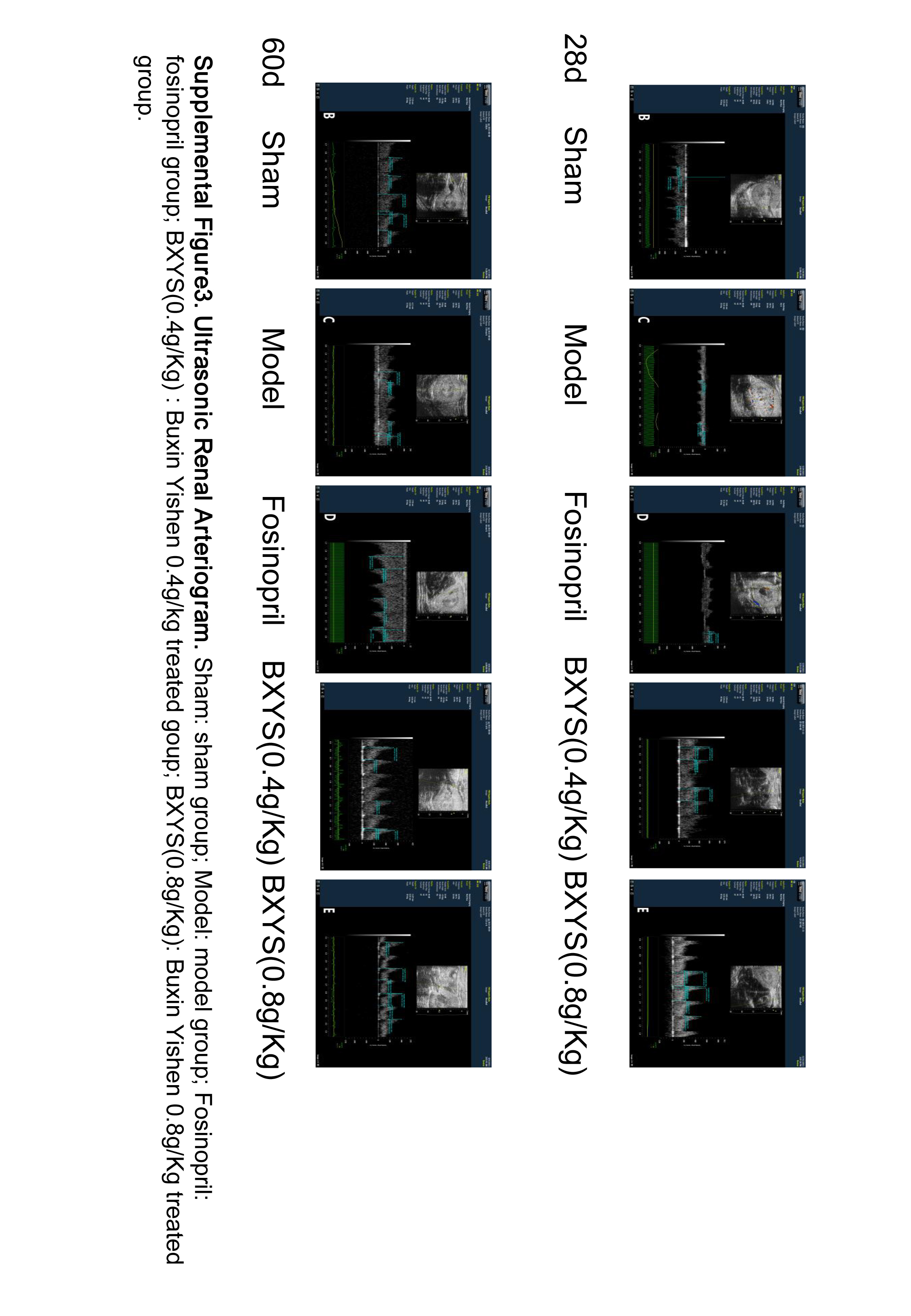

Supplement: Supplementary file 3 [file Image_3.jpeg]
